# Milk Matters: Soluble Toll-Like Receptor 2 (sTLR2) in Breast Milk Significantly Inhibits HIV-1 Infection and Inflammation

**DOI:** 10.1371/journal.pone.0040138

**Published:** 2012-07-06

**Authors:** Bethany M. Henrick, Kakon Nag, Xiao-Dan Yao, Anna G. Drannik, Grace M. Aldrovandi, Kenneth L. Rosenthal

**Affiliations:** 1 McMaster Immunology Research Centre, Michael G. DeGroote Institute for Infectious Disease Research, Department of Pathology and Molecular Medicine, McMaster University, Hamilton, Ontario, Canada; 2 Children’s Hospital Los Angeles, Saban Research Institute, Departments of Pediatrics and Pathology and Laboratory Medicine, Los Angeles, California, United States of America; University of Cape Town, South Africa

## Abstract

The majority of infants who breastfeed from their HIV-positive mothers remain uninfected despite constant and repeated exposure to virus over weeks to years. This phenomenon is not fully understood but has been closely linked to innate factors in breast milk (BM). Most recently we have focused on one such innate factor, soluble Toll-like receptor 2 (sTLR2) for its significant contribution as an inhibitor of inflammation triggered by bacterial and viral antigens. We hypothesized that sTLR2 in BM inhibits immune activation/inflammation and HIV-1 infection. sTLR2 protein profiles were analyzed in HIV-uninfected BM and showed dramatic variability in expression concentration and predominant sTLR2 forms between women. sTLR2 immunodepleted BM, versus mock-depleted BM, incubated with Pam_3_CSK_4_ lead to significant increases in IL-8 production in a TLR2-dependant fashion in U937, HEK293-TLR2, and Caco-2. Importantly, TLR2-specific polyclonal and monoclonal antibody addition to BM prior to cell-free R5 HIV-1 addition led to significantly (*P<0.01, P<0.001*, respectively) increased HIV-1 infection in TZM-bl reporter cells. To confirm these findings, sTLR2-depletion in BM led to significantly (*P<0.001*) increased HIV-1 infection in TZM-bl cells. Notably, immunodepletion does not allow for the complete removal of sTLR2 from BM, thus functional testing shown here may underestimate the total effect elicited by sTLR2 against HIV-1 and synthetic bacterial ligand. This study provides evidence for the first time that sTLR2 in BM may provide a dual protective role for infants breastfeeding from their HIV-infected mothers by; (1) immunomodulating pro-inflammatory responses to bacterial ligands, and (2) directly inhibiting cell-free HIV-1 infection. Thus, sTLR2 in BM may be critical to infant health and prove beneficial in decreasing vertical HIV-1 transmission to infants.

## Introduction

Breast milk (BM) is unique in its ability to fulfill infant nutritional requirements and, arguably more importantly, protect the newborn from environmental and infectious agents during the early stages of life. This protection comes as a complex milieu of innate and adaptive immune components in BM including maternal antibodies, immune cells and innate antimicrobial and immunomodulatory factors [1]. Importantly, many uncharacterized innate factors in BM have not had their functions fully elucidated, although they undoubtedly cooperate to protect the immunologically naïve infant. Numerous publications, originating in 1935 by Grulee *et al*., [1] have correlated the beneficial role of breastfeeding with significant decreases in infant morbidity and mortality. More contemporary publications show decreased prevalence of specific childhood diseases, including respiratory and gastrointestinal infections, particularly in developing nations [2]–[4], making exclusive breastfeeding (EBF) an optimal option for most mothers. Notwithstanding, when a mother is HIV-positive a dilemma arises since there is a risk of vertical HIV- transmission through BM. Paradoxically, however, a number of cohort studies have shown significantly reduced HIV-transmission rates when HIV-infected mothers exclusively breastfed (EBF) compared to mothers who mixed fed their infants [5]–[8]. This phenomena is not fully understood but may be linked to short-lived innate factors in BM that contribute to both reduced immune activation and inhibition of R5 HIV-1 [9], [10], the predominant phenotype transmitted from mother-to-child [11]–[14]. Importantly, activation of the immune system, namely inflammation, is a strong prognostic marker of HIV infection [15], and has been associated with increased HIV acquisition risk caused by activation in the genital tract due to pre-existing sexually transmitted infections (STI) [16]–[19].

Toll-like receptors (TLRs) are innate sensing molecules, known as pattern-recognition receptors (PRRs), that sense ‘danger’ signals or pathogen associated molecular patterns (PAMPs) [20]. TLR2 has a special place among all TLRs in its well characterized recognition of a large breadth of pathogens, including bacteria, viruses, fungi, mycobacteria, and parasites [21]. In contrast to the majority of TLRs, TLR2 has a soluble form (sTLR2) that is produced through post-translational modification [22], [23]. It was first shown to be present in high concentration in BM [23] and was further described in various natural sTLR2 forms in amniotic fluid and saliva with significant immunomodulatory functions to known TLR2 agonists [22]–[26].

In the present study, we confirm and extend our understanding of BM sTLR2 as an important immunomodulator of PAMP-induced proinflammatory cytokine production in the nursing infant’s intestinal mucosa [24]. Further, we demonstrate, for the first time, that BM sTLR2 plays a dual role in inhibiting R5 HIV-1 infection and inflammation, which may prove important in understanding reduced transmission rates to EBF infants from HIV-infected mothers.

## Methods

### Study Cohort and Breast Milk

 study was approved by McMaster Research Ethics Board (REB Approval #08-176), and the CCI of Children’s Hospital, Los Angeles. All participants provided voluntary written informed consent. Inclusion criteria included breastfeeding women who were HIV-uninfected and did not report complications *in utero* during their full-term pregnancies or *intra partum*. Women were excluded if they had had caesarean sections, their pregnancies were not full-term, or they were diagnosed with mastitis *post partum*. Samples included in these analyses were taken from women who were not taking medications other then vitamin supplements *intra* or *post partum*, and did not receive an epidural *intra partum*. Three mothers reported minor colic and one reported silent reflux in their infants. Milk samples were self-collected into sterile tubes within the first week and at one month, three months and six months postpartum, and immediately shipped on ice for processing in our laboratory. Samples were separated into lipid, supernatant, and cellular fractions and stored at −80°C and liquid nitrogen, respectively. One-month postpartum samples were also collected from women in a Los Angeles cohort. Specific demographics are provided in Table 1. BM supernatant fractions were used for Western blotting or were filter-sterilized (0.45 µm) for functional assays to avoid cellular contamination.

**Table 1:** Characteristics of Healthy HIV-uninfected Women Participants.

**Samples Collected:**				
< one week				13
> two weeks				5
**Median Maternal Age**				31.5
**Mean Maternal Age**				31.78
**Median Parity**				2
**Mean Parity**				2.22
**Clinical Diagnosis of Mastitis**				0
**Racial Background:**				
African American				1
Asian				1
Caucasian				14 (84.6%)
Jamaican				1 (8.3%)
Indian				1 (8.3%)
**Exclusively Breast Feeding:**				
< one week samples				13/13 (100%)
> two weeks				3/5 (60%)

### Cell Lines and Reagents

Human embryonic kidney (HEK293), HEK293-TLR2, and human monocytic U937 cells were cultured in DMEM or RPMI supplemented with 10% FBS (Invitrogen), 10 µM HEPES (Invitrogen), 2 µM L-glutamine (Invitrogen), 100 units/mL penicillin/streptomycin (Sigma-Aldrich), respectively and maintained at 37°C and 5% CO_2_. HEK293-TLR2 additionally required selection media with 10 µg/mL blasticidin. All cell lines were plated to reach 5×10^4^ cells/well for each experiment in 96-well plates. Caco-2 intestinal epithelial cell line were cultured as described previously [27] and plated at 2.5×10^4^ cells/well and grown overnight.

### Western Blot

BM was evaluated for total protein concentration using a NanoVue luminometer (GE Healthcare) and at equal concentration was boiled with 5× Laemmli reducing buffer and resolved in SDS-PAGE as per standard protocol. Signals were developed and detected as previously described [23]. sTLR2 bands were analyzed using Un-Scan-It image digitizing software (Silk Scientific Inc.). Recombinant sTLR2 was used as reference standard.

### Antibodies

The following anti-sTLR2 antibodies were used: goat polyclonal IgG, N-17 (sc-8689); mouse monoclonal IgG2a, TL2.1 (sc-21759), TL2.3 (sc-21760); mouse monoclonal IgG1, T2.5 (sc-52736) (Santa Cruz Biotechnology) and mouse monoclonal IgG1 ascites, 1030A5.138 (ebioscience). Secondary antibodies included: donkey anti-goat IgG and chicken anti-mouse IgG (Santa Cruz). Isotype controls included: goat and mouse (Dako Canada Inc.).

### Immunodepletion of sTLR2 from Breast Milk

Immunodepletion of sTLR2 from mucosal samples using N-17 antibody and Protein G-Sepharose (GE Healthcare) was described previously [22], [23]. Mock-depleted samples were treated simultaneously and with similar reagents without specific antibodies. Cyanogen-bromide (CNBr) was also used for immunodepletion of sTLR2 forms as per manufacturer’s instructions (Pharmacia Biotech). TLR2 antibody N-17 or T2.5 was linked to conjugated CnBr beads as per manufacturer’s instructions. Bead washing up to 10 washes were tested using Western blotting to ensure no free antibody remained in the system (data not shown).

### Immunoassays

ELISA Duoset was used to measure IL-8 levels in cell culture systems according to manufacturer’s instructions (R&D Systems). sTLR2 cytometric bead assay (CBA) was developed in our lab as per manufacturer’s instructions (BD Biosciences). Capture beads were covalently bound with T2.5 Ab and N-17-Phycoerythrin (PE) Ab was used as detection antibody (Santa Cruz). Human IL-8 flex-set (BD Biosciences) was used to determine IL-8 production in Caco-2 supernatants.

### HIV Preparation and Reporter Assay

R5-tropic ADA or BaL virus was prepared and tissue culture infectious dose (TCID) of pooled supernatants as well as *in vitro* infection functional assays were determined using TZM-bl cells as described previously [28]. Briefly, 5×10^4^ cells/well were plated in a 96-well plate with 25 µg/mL of diethylaminoethyl-dextran (Sigma). Luciferase activity from TZM-bl (JC53-BL) cells (kindly provided by Dr. D. Montefiori, Duke University, North Carolina) was measured using Bright-Glo reagents (Promega) and luminescence was read using the Veritas luminometer (Promega) and reported as relative light units (RLU).

### MTT Viability Assay

MTT assay was used to determine viability of TZM-bl reporter cells exposed to human breast milk. The assay was performed using the manufacturer’s instructions (Biotium Inc., CA, USA). TZM-bl cells (5×10^5^) were plated in a 96-well plate, exposed to 2-fold serial dilutions of human breast milk in triplicate, and incubated at 37°C for 24 hours. 10 µl of MTT solution was added and incubated for 4 hours at 37°C. After incubation, the media was discarded and 200 µl DMSO (dimethyl sulfoxide) was added. Optical density (OD) was tested at 570 nm (reference 630 nm) in an ELISA plate reader.

### cDNA Analysis

Total RNA was extracted from intestinal epithelial cells, human Caco-2 and fetal FHs-074, using TRIzol reagent (Invitrogen) following the manufacturer’s instructions. RNA was reverse transcribed. Primers used to amplify TLR2 and 18 S cDNA were described previously [29].

### Statistical Analysis

Data were plotted and analyzed using Prism (GraphPad Software). Non-parametric tests were used including Mann–Whitney U-tests and Student t-test for unmatched comparisons and ANOVA for multiple comparisons. *P* was considered statistically significant if <0.05.

## Results

### Predominant sTLR2 Polypeptides Profile in Breast Milk

**Figure 1:**
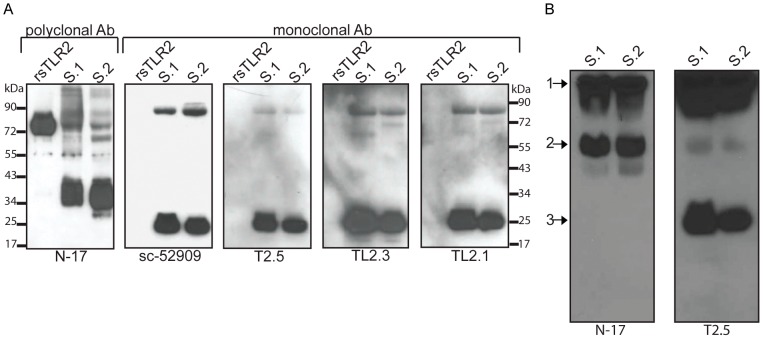
Predominant sTLR2 polypeptides profiles in breast milk. Predominant sTLR2 polypeptide profiles found in multiple breast milk (BM) samples using Western and Native blot analysis. (**A**) BM samples (10 µg total milk protein) with commercial rsTLR2 were evaluated by Western blot analysis with N-17 pAb and 4 mAb (sc-52909, T2.5, TL2.3, TL2.1). pAb N-17 detected commercial rsTLR2 as well as multiple bands in BM; the predominant BM forms were ∼83 kDa and ∼38 kDa sTLR2 forms. In contrast, mAbs did not detect the commercial rsTLR2. In BM mAbs detected the ∼83 kD band, as well as a unique ∼26 kDa sTLR2 form, which was not detected with the N-17 pAb. (**B**) N-17 pAb and T2.5 mAb were used in Native blot analysis of two BM samples. N-17 pAb detected two large proteins (arrow 1 & 2), while T2.5 mAb detected 3 proteins (arrow 1, 2 & 3). A representative data set from three experiments is shown.

We set out to examine the sTLR2 protein profiles in breast milk (BM) samples from healthy HIV-uninfected women using Western and Native blot analysis. Using reducing methods and a variety of TLR-specific antibodies, our data show three major polypeptide bands of ∼83 kDa, ∼38 kDa and ∼26 kDa and additional minor polypeptides of ∼130 kDa, and ∼66 kDa (Fig.1A). These data compliment previously published reports that identified similar sTLR2 polypeptide bands, including ∼83 kDa, ∼66 kDa, ∼38 kDa, and ∼26 kDa [23] under reducing conditions. However, the predominant sTLR2 polypeptide forms described here (namely 38 kDa and 26 kDa) were substantially different in size compared to a previously reported predominant sTLR2 polypeptides in BM (66 kDa) [23]. Additionally, polypeptide band patterns varied among samples tested (Fig.1A). Data indicate the polyclonal antibody (pAb) N-17 was specific for the ∼83 kDa and ∼38 kDa sTLR2 polypeptide bands. Multiple TLR2-specific monoclonal antibodies (mAbs) detected a similar ∼83 kDa band as well as a separate and unique ∼26 kDa band (Fig.1A). Notably, pAb detected the presence of commercially available rsTLR2, while anti-TLR2 mAbs were unable to detect the recombinant protein (Fig. 1A). Further, using Native blot analysis, we identified three predominant sTLR2 protein complexes found in BM (Fig. 1B). Specifically, N-17 and T2.5 Abs could detect identical native proteins indicated by arrow 1 and 2. Additionally, T2.5 Ab detected a unique sTLR2 protein identified with arrow 3, which may be specific for a C-terminal portion of TLR2 extracellular domain as has been previously suggested [30].

### Variation in sTLR2 Polypeptides between Different Women

**Figure 2:**
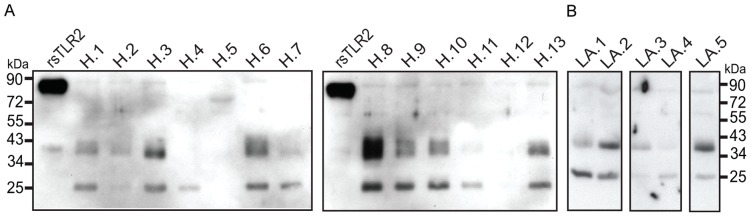
Variation in sTLR2 polypeptides between different women. Breast milk samples (10 µg total protein) from HIV-uninfected women were evaluated using a cocktail of anti-sTLR2 antibodies (N-17 pAb and T2.5 mAb) using Western blot analysis. Results show dramatic variation in predominant sTLR2 polypeptide (∼38 kDa and ∼26 kDa) expression in milk from different women. (A) Samples taken within one week postpartum. (B) Samples taken within one month postpartum.

Multiple BM samples collected within one week (N = 13) and at one month (N = 5) post partum were tested under reducing conditions to further investigate the extent of sTLR2 polypeptide variation among women. Two major observations were noted: (1) The intensities of the two predominant sTLR2 bands were remarkably variable, showing high (samples H3–6, H8–10, and H13), intermediate (H1, H11, LA1, LA2, and LA5) and very low (H2, H7, H12, LA3, and LA4) levels of sTLR2 expression despite equivalent amount of protein for each sample; (2) sTLR2 forms varied considerably among different women’s BM. Under our detection condition, the 38 kDa polypeptide band appeared at a substantially greater concentration than the 26 kDa sTLR2 polypeptide band in about half the women (samples H3, H6, H8–10, H13, LA2, LA3, and LA5) while in approximately 25% of BM samples higher concentrations of 26 kD were observed (samples H1, H11, LA1, and LA4) (Fig. 2).

### Specificity of Anti-sTLR2 Antibodies

**Figure 3:**
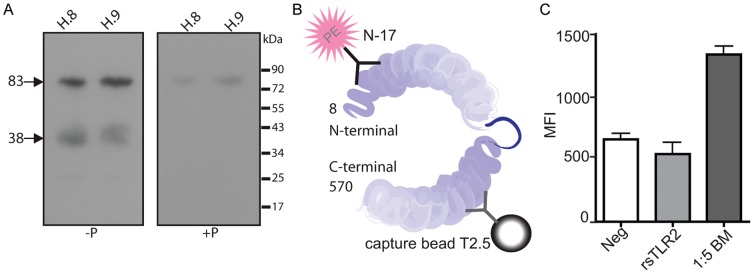
Specificity of anti-sTLR2 antibodies. The specificity of anti-sTLR2 antibodies was confirmed using a peptide competition assay and CBA. (A) N-17 was preincubated without (-P) or with (+P) 5× molar excess of peptide (N-17P) prior to immunoblotting. Pre-incubation with excess peptide markedly reduced both the ∼83 kDa and ∼38 kDa isoforms of sTLR2. Results representative of breast milk (BM) samples from different donors tested. (B) Schematic of cytometric bead array shows that beads coated with T2.5 mAb pulled natural sTLR2 out of milk and were detected with phycoerythrin (PE) labeled N-17 detection antibody. (C) CBA analysis of commercial rsTLR2 and BM dilution clearly shows system can detect natural sTLR2 but cannot detect commercial rsTLR2. A representative data set from triplicate experiments is shown.

In light of these observations, we evaluated the specificity of anti-TLR2 Abs. N-17 specificity was confirmed using a competition assay with the corresponding peptide (N-17P), shown previously [22], [26]. Pre-incubation of pAb with N-17P negated detection of the 38 kDa, and a substantial amount of 83 kDa polypeptide bands in BM when evaluated using N-17 Ab (Fig. 3A). To build upon previous reports confirming the specificity of mAb [25], [30], [31], we used a sandwich cytometric bead array (CBA) (Fig. 3B) designed in our laboratory. A bead complex using a capture antibody (mAb T2.5) and detection antibody (pAb N-17-PE) confirmed mAb specificity for sTLR2 in BM. However, the CBA did not detect commercially available recombinant extracellular TLR2 (rsTLR2) (Fig. 3C), which was consistent with our TLR2 profiling Western blot data (Fig. 1).

### Expression Kinetics, Source, and Bioavailability of sTLR2 Expression

**Figure 4:**
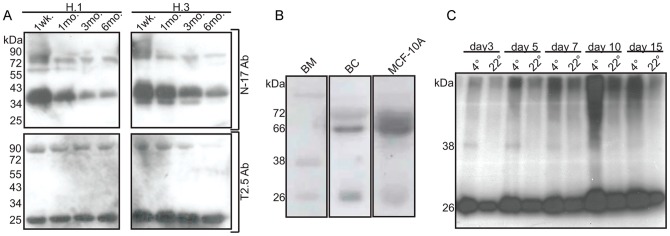
Expression kinetics, source, and bioavailability of sTLR2. sTLR2 expression kinetics, source and bioavailability in breast milk (BM) (10 µg total milk protein) were evaluated using Western blot analysis. (**A**) BM evaluated at one week to six months using N-17 pAb (top panel) and T2.5 mAb (bottom panel) indicated a reduction of the ∼38 kDa and consistent expression of the ∼26 kDa sTLR2 polypeptide postpartum. A representative data set from four different donors is shown. (B) BM, BM cells (BC), and MCF-10A supernatant were tested. ∼38 kDa sTLR2 was observed in BM, but absent from BC and MCF-10A. ∼26 kDa sTLR2 form was observed in BM, BC, and MCF-10A. (C) BM stored at 4°C or 24°C revealed ∼38 kDa sTLR2 polypeptide was quickly degraded compared to the slight degredation of the ∼26 kDa form. Western blot analyses were developed with a cocktail of N-17 pAb and T2.5 mAb. A representative data set from triplicate experiments is shown.

Western blot analyses of BM collected at different time points following delivery revealed sTLR2 expression is regulated postpartum since the expression of the 38 kDa decreased gradually (Fig. 4A, top panel), thus complimenting previous literature showing decreases in major sTLR2 polypeptides post partum [22], [23]. In contrast, the expression of the 26 kDa polypeptide remained unchanged over six months (Fig. 4A, bottom panel).

To determine the source of sTLR2 polypeptides, supernatants collected from BM cells and a mammary epithelial cell line, MCF-10A were examined. Our results revealed that the 26 kDa polypeptide is, at least in part, produced by the MCF-10A and BM cellular fraction (Fig. 4B). Surprisingly, however, 38 kDa sTLR2 was not detected in these cell supernatants (Fig. 4B). These findings lead to two possibilities–(1) the 38 kDa form is not produced by the cells tested here or (2) the protein is not stable in cell culture conditions. Therefore, we next tested the stability of 38 kDa and 26 kDa sTLR2 in BM over a 15-day period, and observed that the 38 kDa form of sTLR2 was not detectable when stored at room temperature (24°C), yet was detectable to 7 days when stored at 4°C (Fig. 4C). Conversely, the 26 kDa sTLR2 was detectable at all time points (Fig. 4C) indicating that 38 kDa is less stable then 26 kDa sTLR2 in BM.

### sTLR2-mediated Augmentation of Pro-inflammatory Cytokines during Bacterial Lipoprotein Exposure

**Figure 5:**
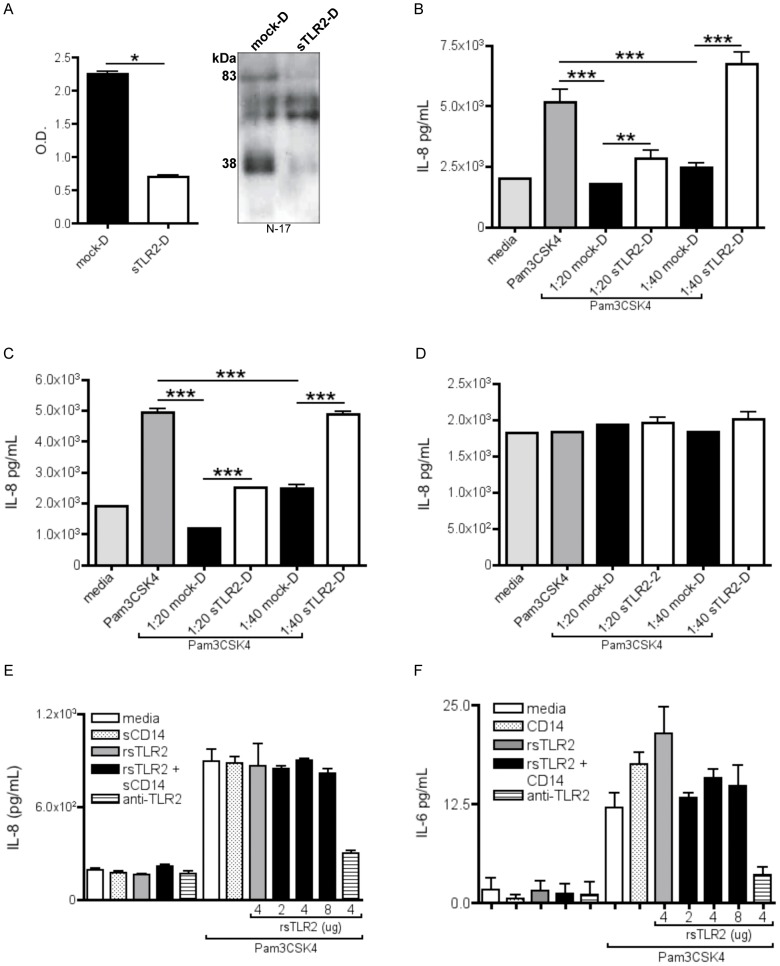
sTLR2-mediated augmentation of pro-inflammatory cytokines during bacterial lipoprotein exposure. (A) Immunodepletion of ∼38 kDa sTLR2 in breast milk (BM) described in materials and methods. Quantitative analysis using TLR2 ELISA indicated a significant (*P<0.05)* decrease in sTLR2-depleted (sTLR2-D) compared to mock-D breast milk (BM). Western blot analysis revealed that ∼38 kDa and ∼83 kDa sTLR2 were markedly reduced compared to mock-D BM. (B-D) 500 ng/mL Pam_3_CSK_4_ was incubated with media or BM that was either mock-D or sTLR2-D for 1 hr at 37°C before being placed on cells. Supernatants were collected for IL-8 ELISA after 18 hours. Results represent (B) U937, (C) HEK293-TLR2 and (D) HEK293. (E) Commercial rsTLR2 was used at varying concentration with or without sCD14 and showed no inhibition of IL-8 or (F) IL-6 production in HEK293-TLR2 cells. Significant increases in pro-inflammatory cytokine, IL-8, was observed in sTLR2-D compared to mock-D BM. *P<0.05, **P<0.01, ***P<0.001. Errors bars, SEM. A representative data set from triplicate experiments is shown.

sTLR2 is an established inhibitor of pro-inflammatory responses to a variety of microbial pathogens (reviewed in [32]). In order to test the functional role of BM sTLR2, we immunodepleted natural forms of sTLR2 from BM using TLR2-specific antibodies and tested prototypic inflammatory cytokine, IL-8, production in cell lines that endogenously express TLR2, including U937 [33] and HEK293-TLR2 in response to the synthetic bacterial lipoprotein, Pam_3_CSK_4_. Qualitative and quantitative evaluation of sTLR2 immunodepletion using Western blot and ELISA analysis, respectively, indicate that a significant amount of sTLR2 was depleted from sTLR2-depleted (sTLR2-D) compared to mock-depleted (mock-D) BM (Fig. 5A). Further, digital semi-quantification analysis revealed that roughly 75% of 83 kDa and 71% of 38 kDa sTLR2 were depleted from BM.

As postulated, sTLR2-D BM did not significantly inhibit the Pam_3_CSK_4_-induced production of pro-inflammatory IL-8 compared to the mock-D BM in U937 and HEK293-TLR2 (1∶20 dilution: *P<0.01, P<0.001;* respectively), in a dose-dependent manner (1∶40 dilution: *P<0.001, P<0.001,* respectively) (Fig. 5B & C). Importantly, HEK293 cells, which are devoid of membrane bound TLR2 [34], demonstrated no significant differences in IL-8 production among groups (Fig. 5D). These data indicate that inhibition of Pam_3_CSK_4_-induced pro-inflammatory response by BM is largely mediated by membrane-bound TLR2.

We next examined the use of recombinant sTLR2 (rsTLR2) in inhibiting pro-inflammatory cytokine production in the HEK293-TLR2 cell line in response to TLR2 ligand, Pam_3_CSK_4._ Multiple reports demonstrate cellular responses against microbial agonists triggered through TLR2 are extremely low without co-receptor sCD14 [35], [36]. Therefore, we demonstrate that at varying concentrations, with or without sCD14, rsTLR2 was unable to reduce IL-8 or IL-6 production (Fig. 5 E & F, respectively). These data precluded the use of recombinant sTLR2 in further *in vitro* experiments.

### sTLR2 Forms Function Similarly in Human Intestinal Epithelial Cells

**Figure 6:**
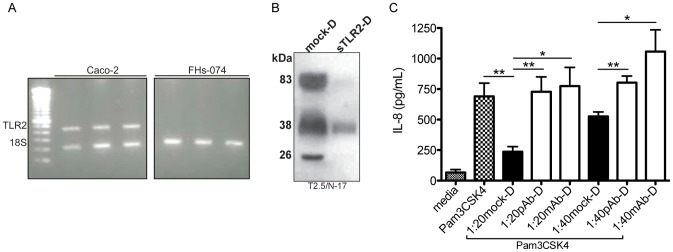
sTLR2 forms function similarly in human intestinal epithelial cells. (A) PCR analysis indicated that Caco-2, intestinal epithelial cells (IEC), expressed TLR2 mRNA while FHs-074 IEC did not. (B) Western blot analysis indicated that the majority of ∼83 kDa and ∼26 kDa sTLR2 forms were removed from sTLR2-depleted (sTLR2-D) compared to mock-D breast milk (BM). (C) *In vitro* testing of Caco-2 cells pre-incubated with mock-D BM for 1 hour at 37°C significantly inhibited IL-8 production (1∶20 *P<0.001;* 1∶40 *P<0.001)* following exposure to Pam_3_CSK_4_ (5 ng/mL). A significant IL-8 increase was observed in pAb-D (N-17 pAb) BM (*P<0.01)* and mAb-D (T2.5) BM (*P<0.05*) following Pam_3_CSK_4_ exposure. **P<0.01, ***P<0.001. Errors bars, SEM. A representative figure of the experiment completed in triplicate is shown.

Given the key role BM has on the development and function of the neonatal gut (reviewed in [37]), the established role of TLR2 in the preservation of intestinal epithelial cell (IEC) barrier integrity [27], [38], as well as our data showing multiple forms of sTLR2 in BM (Fig. 1), we considered the functional role of predominant sTLR2 polypeptides on human IEC in response to known TLR2 ligand, Pam_3_CSK_4_. Caco-2, a well-characterized IEC line that expresses TLR2 shown here (Fig. 6A) and elsewhere [39], was chosen since an infant IEC cell line is not commercially available. Fetal intestinal epithelial cell line (FHs 074) was considered for use, however proved unsuitable, as TLR2 cDNA was not detected (Fig. 6A).

Western blot analysis shows that the majority of sTLR2 was removed using T2.5 mAb immunodepletion (Fig. 6B). Optical densitometry confirmed that predominant sTLR2 forms were depleted in sTLR2-D compared to mock-D BM by the following percentages: 95% of 83 kDa, 64% of 38 kDa, and 99% of 26 kDa form in this batch preparation (Fig. 6A). The 38 kDa appears partially immunodepleted along with the majority of 83 kDa and 26 kDa forms, and may be due to the co-identification of two sTLR2 forms with both antibodies as shown previously in Native blots (Fig. 1B). Similar to experiments shown previously, mock-D BM, pAb-D, or mAb-D BM was incubated with Pam_3_CSK_4_ prior to addition to the IECs. As postulated, pAb-D and mAb-D BM incubated with Pam_3_CSK_4_ lead to significantly (*P<0.01, P<0.05,* respectively) increased IL-8 production compared to mock-D BM. These data indicate that predominant sTLR2 forms are involved in inhibiting Pam_3_CSK_4_-induced inflammation in IECs.

### sTLR2-significantly Inhibits HIV-1 Infection in Reporter Assay

The majority of infants breastfed by HIV-positive mothers remain uninfected despite constant and repeated exposure to virus over weeks, months or even years [5]–[8]. Given the large quantity of sTLR2 in BM observed in our laboratory as well as others [23], and the role of TLR2 in sensing and activating anti-viral responses to a number of viruses [40]–[44], we hypothesized that BM sTLR2 may play a role in inhibiting HIV-1 infection.

**Figure 7:**
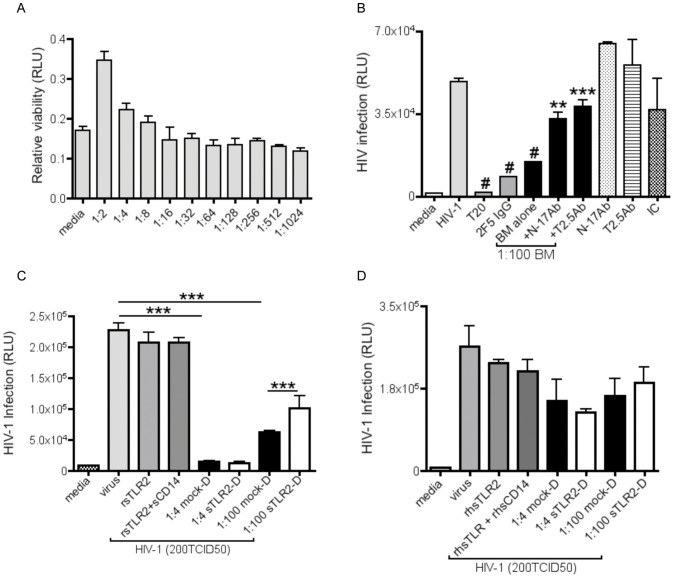
sTLR2 significantly inhibits HIV-1 infection in reporter assay. (A) MTT assay indicates that HIV-uninfected breast milk (BM) was not toxic to TZM-bl cells. (B) HIV-uninfected BM was incubated with either N-17 pAb or T2.5 mAb (200 before R5 HIV-1 (200 TCID_50_) and then placed on TZM-bl cells. T20, 2F5IgG, and 1∶100 BM significantly inhibited infection (*P<0.001)*. A significant increase (*P<0.001, 0.01,* respectively*)* in HIV-1 infection was shown when sTLR2-specific N-17 or T2.5 Ab were pre-incubated with BM. N-17 pAb and T2.5 mAb alone and isotype control (200 ng/mL) did not inhibit HIV-1 infection. (C) rsTLR2+/− sCD14 or pooled HIV-uninfected mock-D or sTLR2-D BM (described in materials and methods) were incubated with R5 HIV-1 (200 TCID_50_) before addition to TZM-bl cells for 48 hours. A significant decrease (*P<0.001*) in HIV infection was observed in cells exposed to mock-D BM. However, a significant increase (*P<0.001*) in HIV infection was detected with sTLR2-depleted BM. (D) rsTLR2+/− sCD14, mock-D, or sTLR2-D was incubated with cells for 1 hour at 37°C, washed with PBS, and then exposed to R5 HIV-1 (200 TCID_50_) did not alter HIV-1 infection. ** P<0.01, ***P<0.001. Errors bars, SEM. A representative data set from four experiments is shown.

We first determined that BM was not toxic to TZM-bl cells (Fig. 7A). To test our hypothesis, TLR2-specific mAb or pAb was incubated with BM prior to the addition of R5 virus and then placed on TZM-bl cells. Our results show that positive controls T20 and 2F5IgG significantly (*P<0.001*) inhibited HIV-1 infection (Fig. 7B) as shown previously [28]. Likewise, 1∶100 BM significantly (*P<0.001*) inhibited HIV-1 infection (Fig. 7B), indicating that 1∶100 BM could be used as a positive control in further experiments, as shown previously [10]. Conversely, when TLR2-specific pAb or mAb were incubated with BM prior to addition of cell-free virus, there was a significant increase (*P<0.*001) in HIV infection (Fig. 7B). Additionally, we found that neither antibodies alone nor isotype controls had any significant effect on HIV-1 infection (Fig. 7B).

To further confirm that sTLR2 was involved in inhibiting HIV-1, we incubated mock-D or sTLR2-D BM with virus prior to being placed on TZM-bl cells. Our results indicated that our positive control, mock-D BM, significantly decreased cell-free HIV-1 infection at a 1∶100 dilution (*P<0.001*) (Fig. 7C), which is consistent with a previous publication [10]. However, when BM was depleted of sTLR2, cell-free HIV-1 infection was significantly increased (*P<0.001)*. Conversely, rsTLR2 that was tested with or without sCD14 did not potently inhibit cell-free HIV-1 infection (Fig. 7C & D). Further, we examined the effect of pre-incubating TZM-bl cells with either mock-D or sTLR2-D BM prior to adding virus, and found no significant difference in HIV-1 infection (Fig. 7B), which indicates that sTLR2 may directly inhibit cell-free HIV-1.

## Discussion

Here we report on predominant forms of sTLR2 in breast milk (BM) that vary between women and show that they have direct effects on inflammatory responses and HIV-1 infection. Results confirm previous findings that indicate sTLR2 is critical in suppressing inflammatory responses to bacterial PAMPs [22], [23], [25], [26], and extend to show direct inhibition of cell-free R5 HIV-1 infection. Thus, sTLR2 may be an important innate factor that protects infants breastfeeding from HIV positive mothers by (1) directly inhibiting cell-free R5 HIV-1 infection, and (2) inhibiting immune activation, namely inflammation of the nursing infant’s gut.

To our knowledge, this is the first time that the major 38 kDa and 26 kDa sTLR2 polypeptides have been reported as predominant sTLR2 forms in BM, although similar-sized sTLR2 forms have been observed in both amniotic fluid, saliva [22], [26], and cervicovaginal fluid (Henrick et al., unpublished data). Variation in predominant sTLR2 polypeptides between different women’s BM samples may be the result of racial, ethnic or genetic variability, which may explain the differences in predominant sTLR2 forms identified between our cohort and the one described by LeBouder *et al* (2003), despite the similarity in sample collection, times postpartum, processing, and Western blot analysis, including antibodies used. Notably, we provide data that multiple TLR2-specific mAbs were unable to detect commercially available human rsTLR2 indicating important structural, and/or conformational differences that were further associated with altered immune functionality *in vitro*. Indeed, rsTLR2 is produced in mouse myeloma cell lines, which may alter glycosylation patterns as opposed to native proteins, and may prove pivotal given the undoubted importance of N-linked glycosylation in proper sTLR2 synthesis, secretion, and function [45], [46]. These data indicate that caution should be used when interpreting data in which commercially available rsTLR2 is used as a replacement for natural sTLR2 forms. Further, interpretation of our data leads us to believe that intermolecular non-covalent interactions between the predominant sTLR2 polypeptide 38 kDa and 26 kDa are responsible for sTLR2 protein complexes that function in a coherent manner to inhibit bacterial PAMP-induced production of pro-inflammatory cytokine production and R5 HIV-1 infection.

Further, experiments into the regulation of major sTLR2 polypeptides in BM may be important in fully appreciating the intricacies of intestinal microbiome development in infants. Our observations indicated that multiple cells of the mammary gland and BM contribute to the generation of 26 kDa sTLR2, however the source of the 38 kDa form remains elusive. Data indicate that 38 kDa sTLR2 production is down-regulated over time postpartum and is susceptible to degradation at room temperature. These observations may correlate with the infant’s maturing immune system [47], and assist in the development of tolerance as well as proper IEC epithelial barrier function [27], [38].

The function of BM sTLR2 has been shown to be immunomodulatory by providing direct attenuation of signaling through membrane TLR2 while still allowing for effective elimination of the pathogen in a mouse model [25]. Here, we demonstrated that sTLR2 in BM has dual utility by providing both anti-inflammatory and anti-viral functions: (1) sTLR2 depleted BM led to significantly increased IL-8 pro-inflammatory cytokine production in U937, HEK293-TLR2 and human IECs exposed to PAM_3_CSK_4_; (2) Inhibition of sTLR2 through the addition of TLR2-specific antibodies as well as sTLR2-depleted BM led to significantly increased HIV-1 infection in TZM-bl reporter assays. To the best of our knowledge, this is the first study to demonstrate that sTLR2 in BM can directly inhibit cell-free R5 HIV-1 infection. However, previous studies have demonstrated a strong correlation between decreased sTLR2 concentrations and HIV-1 progression [48], and high levels of TLR2 expression on monocytes taken from HIV-1 infected patients [49]. Indeed, there are numerous publications indicating that many viruses including measles, VSV, CMV, hepatitis C and herpes virus activate TLR2 through viral glycoproteins leading to the production of proinflammatory cytokines [40]–[44], [50]. Further, we previously showed significantly increased TLR2 expression in peripheral blood mononuclear cells of Kenyan commercial sex workers with AIDS, which is reduced with HAART treatment [29]. Our data is timely given a recent publication confirming increased levels of HIV-1 RNA in BM positively correlate with vertical transmission [51], [52].

Notably, immunodepletion does not allow for the complete removal of sTLR2 from BM, thus functional testing shown here may underestimate the total effect elicited by sTLR2 against HIV-1 and synthetic bacterial ligand. Likewise, this technique has experimental limitations that do not preclude the possibility that an unknown binding partner of sTLR2 may also contribute for the reduction of pro-inflammatory cytokine production in response to bacterial ligands and HIV-1 inhibition shown here. Future research is warranted to determine the exact mechanism of sTLR2 functionality including the involvement of cofactor CD14 in the direct binding and inhibition of HIV, as the necessity of the cofactor differs between viral agonists [40], [42], [50].

In conclusion, the current study provides evidence for the first time that sTLR2 in BM may provide a dual protective role for infants breastfeeding from their HIV-infected mothers. We confirm that BM sTLR2 complexes inhibit production of pro-inflammatory cytokines during bacterial ligand exposure, and extend the functional role of BM sTLR2 complexes to include inhibition of cell-free R5 HIV-1 infection. As discussed previously, immune activation has marked effects on HIV-1 acquisition (reviewed in [15]), therefore, data shown here may prove critical in understanding vertical HIV-1 transmission through BM, and indicates sTLR2 may be an important factor in a complex milieu of immunomodulatory and anti-viral factors that help protect the majority of infants from becoming infected despite repeated and prolonged exposure to HIV-1 infected BM.
